# Rare inheritance of Leri-Weill Syndrome due to crossover of short stature Homeobox Gene (SHOX) Deletions between X and Y Chromosomes: a case report

**DOI:** 10.1186/1687-9856-2013-11

**Published:** 2013-06-28

**Authors:** Marisa Censani, Kwame Anyane-Yeboa, Ronald Wapner, Erica Spiegel, Edwin Guzman, Sharon E Oberfield

**Affiliations:** 1 Department of Pediatrics, Division of Pediatric Endocrinology, Columbia University Medical Center, PH5E-522, 622 West 168th Street, New York, NY 10032, USA; 2 Department of Pediatrics, Division of Clinical Genetics, Columbia University Medical Center, 3959 Broadway, 6 N-601A, New York, NY 10032, USA; 3 Department of Maternal Fetal Medicine, Columbia University Medical Center, 3959 Broadway, 12 Central, Room 1207, New York, NY 10032, USA; 4 Columbia University Medical Center, 622 West 168th Street, PH 5E-522, New York, NY 10032, USA

**Keywords:** Leri-Weill syndrome, Madelung deformity, Pseudoautosomal region 1, Short stature, SHOX

## Abstract

**Background:**

Leri-Weill syndrome (LWS) is a genetic disorder caused by deletions or mutations in the SHOX gene or by deletions downstream of the gene and is classically characterized by short stature, mesomelic shortening of forearms and legs, and Madelung deformity. Correct identification of short stature homeobox-containing gene (*SHOX*) deficiency in children with growth problems is vital for appropriate initiation of growth hormone therapy.

**Method:**

We report a phenotypically normal 23 day old male infant born to a father diagnosed with Leri-Weill syndrome at age 12 years with a documented SHOX deletion on his X chromosome. The patient’s fetal long bones had been found to be about three weeks delayed in growth on prenatal ultrasound during the second trimester.

**Results:**

The infant underwent genetic evaluation at 23 days of life and was found to have a SHOX deletion on Yp11.32 identified using single nucleotide polymorphism microarray (SNP) analysis and confirmed by FISH using a SHOX gene probe.

**Conclusion:**

We report the case of a male infant diagnosed with Leri-Weill syndrome with an unusual documented inheritance between father and son due to crossover between X and Y chromosomes during *paternal* meiosis. Our case is the youngest patient in literature documented by FISH analysis to have an X to Y chromosome transfer and the first of these patients diagnosed *prior* to onset of short stature or Madelung deformity. Our patient was identified prior to growth failure and can now be monitored for growth abnormalities with the ability to implement growth augmentation therapy without delay. Our case highlights the importance of advising affected SHOX patients of risks to future offspring and supports screening off-spring of parents carrying SHOX abnormalities regardless of sex.

## Background

Short stature homeobox-containing gene (SHOX) is a growth regulating gene present on the pseudoautosomal region 1 (PAR1) on the distal end of the X and Y chromosomes. SHOX haploinsufficiency is considered in the differential diagnosis for short stature in children and is currently an FDA approved indication for growth hormone therapy. Leri-Weill syndrome (LWS) is a genetic disorder caused by deletions or mutations in the SHOX gene or by deletions downstream of the gene. It is classically characterized by short stature, mesomelic shortening of forearms and legs, and Madelung deformity [1]. We present the rare case of a male infant diagnosed with Leri-Weill syndrome in which an originally X-located SHOX deletion from father was transmitted to son’s Y chromosome by crossover during meiosis. We also review the literature to date and discuss the future implications of such findings.

## Case presentation

The male index patient (IV: 1, Figure 1) was the first child of Caucasian non-consanguinous parents. He was born at 39½ weeks gestation with a normal birth weight of 2.95 kg, (25^th^ centile), and length 50 cm (50^th^ centile). On prenatal ultrasound, fetal long bones were found to be about three weeks delayed in growth during the second trimester. The infant underwent genetic evaluation at 23 days of life since the father had been previously diagnosed with Leri-Weill syndrome with a documented SHOX deletion on his X chromosome del (X)(p22.33p22.33) confirmed by FISH using a SHOX gene probe. The father’s deletion was confirmed with a FISH probe specific for the SHOX gene. On examination, the patient measured 51.5 cm (25-50th centile) and weighed 3.45 kg (10-25^th^ centile) with head circumference of 36 cm (25^th^ centile). Although physical examination was unremarkable, a DNA microarray study was performed to rule out the presumed extremely small possibility of the deletion crossing over to the Y chromosome of the index patient (IV: 1, Figure 1).

**Figure 1:**
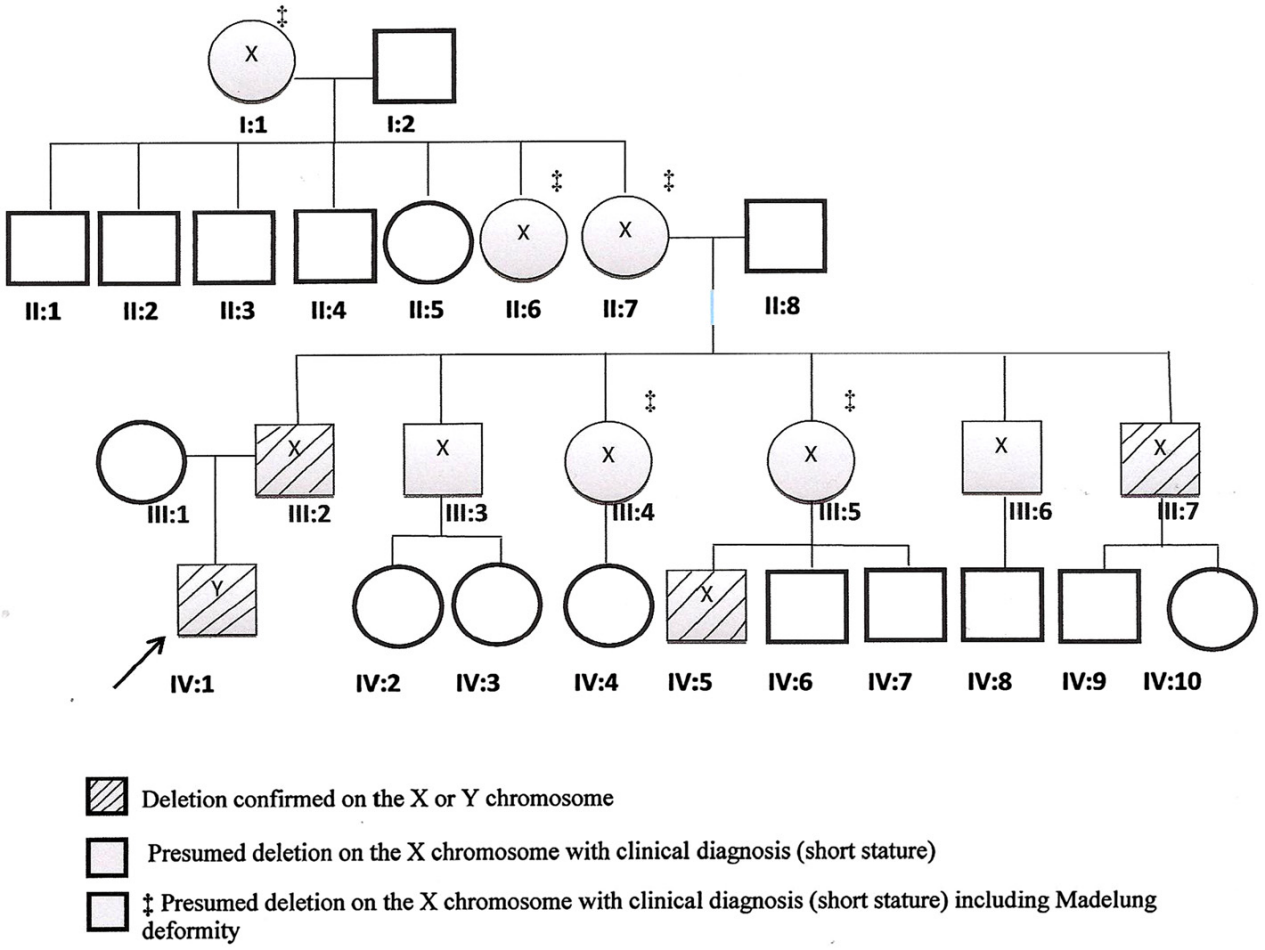
Pedigree of the family.

The mother (III:1) was of normal height and stature at 154.9 cm tall. The father (III:2) was of short stature at 157.5 cm tall. The father had been diagnosed with Leri-Weill syndrome at 12 years of age, and had not been treated with growth hormone. His past medical history was also notable for an insulinoma diagnosed at 8 years of age with a partial pancreatectomy at 18 years. Three paternal uncles, two paternal aunts, one paternal cousin and patient’s paternal grandmother, in addition to patient’s paternal great aunt, and paternal great-grandmother, were also noted to be affected with LWS (see Figure 1).

A SHOX deletion of 262 Kb on Yp11.32 was identified using single nucleotide polymorphism microarray (SNP) analysis in the patient and confirmed by FISH using a SHOX gene probe.

Our infant patient was diagnosed with Leri-Weill syndrome resulting from an unusual documented inheritance between father and son due to crossover of the SHOX deletion between X and Y chromosomes during *paternal* meiosis. We report the youngest patient in literature documented by FISH analysis to have an X to Y chromosome transfer of an originally X-located SHOX deletion. The mechanisms resulting in SHOX deficiency include gene mutations and whole gene deletions of the pseudoautosomal region1 (PAR1) of differing sizes [2,3]. Approximately two-thirds of individuals with SHOX mutations have large scale SHOX deletions that vary in size between 90 kb and 2.5 Mb or more. Point mutations comprise the remaining one-third of SHOX mutations causing SHOX-related haploinsufficiency [4]. Since SHOX is located in the pseudoautosomal regions 1 (PAR1) present on both the X and Y chromosomes, mutations within these genes may segregate independent of sex and be inherited in an autosomal fashion, termed pseudo-autosomal dominant inheritance [2].

Transfer of the deleted or mutated SHOX gene to the alternate sex chromosome due to crossover during meiosis has been described. However, most reports discussed recombination events in the PAR1 in the context of a Y-located SHOX deletion transmitted from father to daughter or included pedigrees that did not differentiate between Y- and X- chromosomal SHOX mutations [5-8]. Indeed, to our knowledge, the transfer of an originally X-located SHOX deletion to the Y chromosome after transmission from father to son has only been documented by FISH in two patients in the literature to date, and *not* in an apparently phenotypically normal male child [9,10].

In a study by Ross *et al.*, subjects were referred for short stature or Madelung deformity and 17 unrelated families with LWD with complete gene deletion in 33 subjects were identified. An X to Y chromosome transfer was mentioned in an 8.3 year old male from his father, with the child’s height noted to be at −2.5 SDS [9]. Musebeck *et al.* analyzed the frequency of SHOX deletions in short stature children and found 5 patients with deletions, one of whom was an 11.75 year old male with a height of 139 (−1.4 SDS) found to have a transfer of the X-located SHOX deletion from his father to his Y chromosome [10]. The 7 month old infant described in our case is the youngest patient in literature documented by FISH analysis to have an X to Y chromosome transfer and is the first patient of these three cases presenting *prior* to onset of short stature or Madelung deformity.

This crossover of SHOX between sex chromosomes is particularly important in the context of genetic counseling. Since 2006, growth hormone (GH) therapy has been an FDA approved indication for treatment of short stature for patients with SHOX deficiency. Leri-Weill syndrome caused by deletions or mutations of the SHOX gene is known to be clinically highly variable [11]. Correct identification of SHOX deficiency in children with growth problems is vital to the implementation of GH therapy in a timely manner.

## Conclusion

Our case highlights the importance of advising affected SHOX patients of risks to future offspring and screening off-spring of parents carrying SHOX abnormalities regardless of sex. Patients should be informed of the possibility that a father carrying a SHOX mutation on the X chromosome can transmit this mutation not only to a daughter but to a son as well due to crossing over between the pseudo-autosomal regions of the X and Y chromosomes during paternal meiosis, albeit a rare occurrence. This finding can also occur in mothers with SHOX mutations on X chromosomes transmitting mutations to the Y chromosomes of their sons. Our case contributes to the knowledge regarding meiotic crossover of the SHOX gene region between the X and Y chromosomes and brings attention to an unusual documented inheritance between father and son. Our patient was identified prior to growth failure and can now be monitored for growth abnormalities with the ability to implement growth augmentation therapy without delay.

## Consent

Written informed consent was obtained from the patient’s father for publication of this case report and any accompanying images. A copy of the written consent is available for review by the Editor-in-Chief of this journal.

## Abbreviations

SHOX: Short stature homeobox-containing gene; LWS: Leri-Weill syndrome; PAR1: Pseudoautosomal region 1.

## Competing interests

The authors declare that they have no competing interests.

## Authors’ contributions

All authors have made significant intellectual contributions to the manuscript. All authors have contributed to the concept and design of the case report. SEO, KA, RW, ES, EG, and MC have diagnosed and/or treated the patient and/or mother. MC, SEO and KA drafted the manuscript, and all authors have critically revised the manuscript for important intellectual content. All authors have read and given approval of the final manuscript version to be published.
